# Bilateral Non-Hodgkin's Lymphoma of the Temporal Bone: A Rare and Unusual Presentation

**DOI:** 10.1155/2016/2641876

**Published:** 2016-12-26

**Authors:** Sanjay Vaid, Jyoti Jadhav, Aparna Chandorkar, Neelam Vaid

**Affiliations:** ^1^Head and Neck Imaging Division, Star Imaging and Research Center, Pune 411001, India; ^2^Department of Otorhinolaryngology, KEM Hospital, Pune 411011, India

## Abstract

Primary lymphoma of the temporal bone is an unusual finding in clinical practice and bilateral affection is even more rare. To the best of our knowledge, there are no reports of bilateral primary temporal bone lymphoma without middle ear involvement in the English medical literature so far. We report, for the first time, a case of primary lymphoma involving both temporal bones which presented with left-sided infranuclear facial palsy. A combination of contrast enhanced magnetic resonance imaging (MRI) and high resolution computed tomography (HRCT) was used to characterize and to map the extent of the lesion, as well as to identify the exact site of facial nerve affection. An excision biopsy and immunohistochemistry revealed diffuse large B-cell non-Hodgkin's lymphoma (DLBCL). Whole body fluorodeoxyglucose (FDG) positron emission tomography-computed tomography study (PET-CT) was performed to stage the disease. The patient was treated with chemotherapy and radiation therapy and is now on regular follow-up. The patient is alive and asymptomatic without disease progression for the last twenty months after initial diagnosis.

## 1. Introduction

Malignancies of the temporal bone are rare with an incidence of less than 0.2% [1, 2] amongst all head and neck cancers. Non-Hodgkin's lymphoma (NHL) is the second most common malignancy found in the head and neck region after squamous cell carcinoma [3, 4]. Involvement of the temporal bone as part of generalized lymphoma has been reported [5, 6]; however, primary involvement of temporal bone without systemic involvement is extremely rare [7]. High resolution multiplanar CT and MRI were useful in demonstrating the local infiltration into overlying soft tissues as well as extension to intracranial compartment. Facial nerve entrapment within the extracranial soft tissue was also well demonstrated using the imaging modalities. Early diagnosis made after an excision biopsy and immunohistochemistry work-up enabled prompt initiation of chemotherapy followed by radiation therapy. Follow-up imaging (MRI and PET-CT) revealed significant regression of the pathology.

## 2. Case Presentation

**Figure 1:**
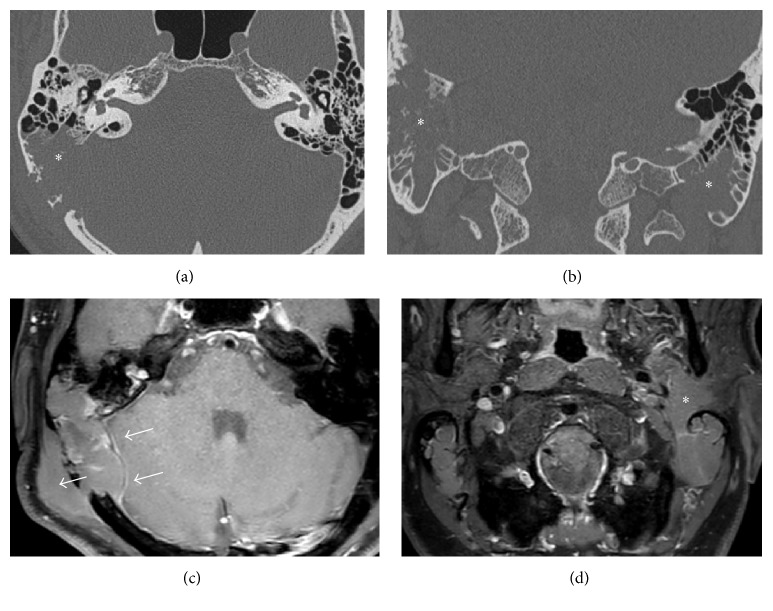
Axial (a) and coronal (b) HRCT images reveal extensive permeative destructive lesions involving both temporal bones (white asterisks). Axial postcontrast fat suppressed T1W axial (c) and coronal (d) images reveal postcontrast enhancement of large associated subgaleal and extracranial intradural soft tissue mass (arrows) on the right side and a moderately large lobulated soft tissue mass along undersurface of left temporal bone (asterisk) causing compression of the extracranial segment of left facial nerve.

A 50-year-old male presented to the Department of Otorhinolaryngology with left facial asymmetry of two months duration. Clinical examination revealed an infranuclear facial palsy on left side with associated bilateral postauricular and occipital region scalp swellings. The scalp swellings were firm and nontender and margins could not be well identified. No neck nodes were palpable on clinical examination. Routine laboratory investigations were within normal limits. The patient was referred to the Department of Imaging to identify the cause of the facial palsy and determine extent of the scalp swellings. A noncontrast HRCT scan of the temporal bones and a contrast enhanced MRI scan of the temporal bones/brain was performed. HRCT of the temporal bones showed extensive irregular permeative osteolytic destruction of the right temporal bone and adjacent right occipital bone. Similar lesions were also noted involving the base of the left temporal bone (Figures 1(a) and 1(b)). HRCT also revealed soft tissue opacification of the mastoid air cells on both sides with erosion of the intercellular septae. Internal and external bony cortical erosions were seen on both sides with erosion of the descending mastoid segment of the left facial nerve canal. The middle and inner ear structures were normal on both sides. MRI scan showed diffuse signal alteration in both temporal bones with associated lobulated, extradural, and subgaleal enhancing soft tissue lesions (Figures 1(c) and 1(d)). The lesions were hypointense on both T1 weighted and T2 weighted images with heterogeneous postcontrast enhancement and showed restricted diffusion on diffusion weighted images (DWI). No calcification or hemorrhagic foci were noted. On the left side, the soft tissue was seen extending along the styloid process into the stylomandibular tunnel up to the deep lobe of parotid gland, involving the extracranial segment of the left facial nerve below the level of stylomastoid foramen. There was no enhancement of the facial nerve seen within the left temporal bone or left internal auditory canal. This finding ruled out retrograde perineural spread of the pathology. On the right side, the intracranial extradural enhancing soft tissue component was seen extending into the middle and posterior cranial fossa. Subgaleal extension of the soft tissue was seen through a defect in the right occipital bone. There was no significant cervical lymph node enlargement detected on the MRI scan. Based on the age of the patient, the clinical presentation, and examination as well as the imaging findings the differential diagnosis included multiple myeloma, metastases, and lymphoma. The clinical presentation and imaging findings were not suggestive of an infective aetiology and hence this diagnosis was not considered.

**Figure 2:**
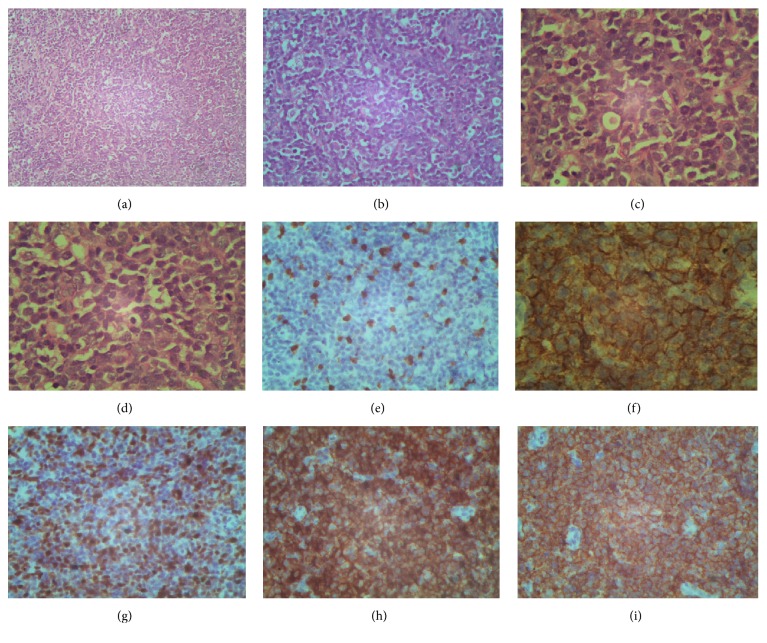
Microscopy showing sheets of medium to large lymphoid cells with hyperchromatic nuclei and scanty cytoplasm (a–d). The cells stained positive for CD3 (e), CD20 (f), Ki67 (g), LCA1 (h), and LCA2 (i).

An excision biopsy of the right subgaleal swelling was performed. Microscopy showed sheets of medium to large lymphoid cells with hyperchromatic nuclei and scanty cytoplasm (Figures 2(a)–2(d)). These cells stained positive for CD3 (Figure 2(e)), CD20 (Figure 2(f)), Ki67 (Figure 2(g)), LCA1, (Figure 2(h)), and LCA 2 (Figure 2(i)) and negative for CyclinD1 (clone Polyclonal), CD5 (Clone 4C7), and CD138 (clone MI-15). The tumor was also positive for Mum-1 and Bcl6 and negative for EBVLMP-1. The above results are suggestive of diffuse large B-cell non-Hodgkin's lymphoma (DLBCL), activated B-cell phenotype. Other blood and bone marrow investigations did not reveal any abnormality.

**Figure 3:**
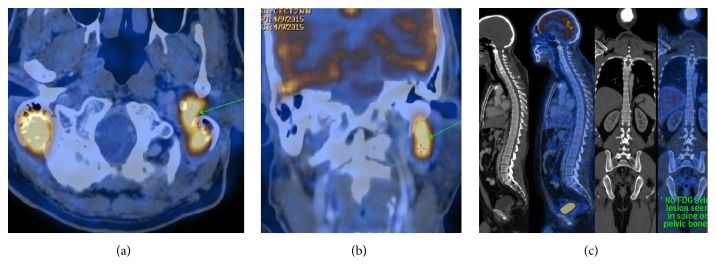
Whole body PET-CT scan revealed FDG-avid lesions (SUV max. 3.6) in both mastoids and along left styloid process (a, b). No other FDG-avid lesions were detected in the rest of the body (c).

A whole body fluorodeoxyglucose (FDG) positron emission tomography-computed tomography study (PET-CT) was performed for staging purposes. The PET-CT scan revealed FDG-avid lesions (SUV max. 3.6) in both mastoids, in the extradural soft tissue mass on the right side and extracranial mass along left styloid process (Figures 3(a) and 3(b)). No other FDG-avid lesions were detected in the rest of the body (Figure 3(c)). This confirmed a primary extranodal involvement of the temporal bones by DLBCL.

**Figure 4:**
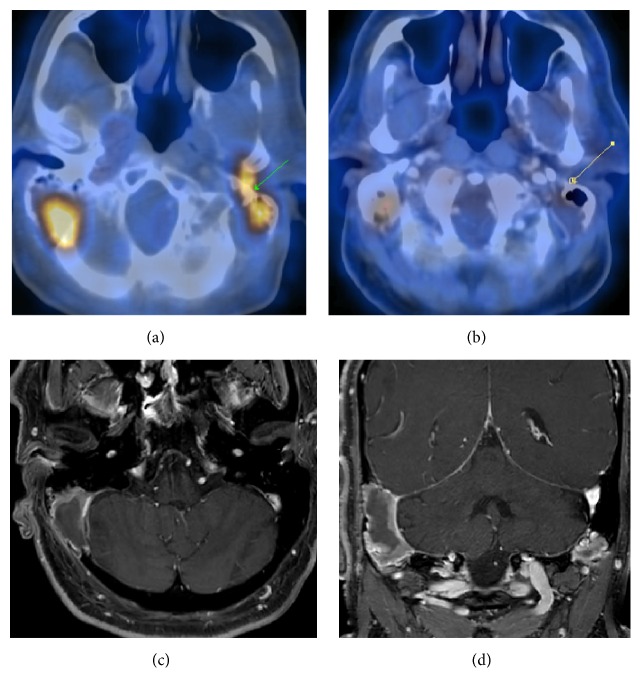
Posttherapy PET-CT scan (a, b) showing significant resolution in the metabolic activity of the mastoid lesions. Contrast enhanced MRI scan (c, d) also showed regression in the size of the enhancing soft tissue masses on both sides.

The patient was treated with six cycles R+CHOP chemotherapy (one cycle of 21 days). This consisted of rituximab at 375 mg/m^2^, cyclophosphamide at 750 mg/m^2^, doxorubicin at 50 mg/m^2^, vincristine at 1.4 mg/m^2^, and prednisolone at 100 mg/m^2^. This was supplemented by prophylactic intrathecal methotrexate at 12.5 mg on day 2 of each cycle and G-CSF (granulocyte colony stimulating factor) at 300 *μ*gm from day 3 to day 7 of each cycle. After six cycles of chemotherapy, patient was treated with six cycles of radiotherapy (45 Gy, 20 fractions). A PET-CT scan was performed three months after the end of radiation therapy which showed significant resolution in the metabolic activity of the mastoid lesions. (Figures 4(a) and 4(b)). The repeat MRI scan also showed regression in the size of the enhancing soft tissue masses on both sides (Figures 4(c) and 4(d)).

Patient also showed signs of clinical improvement and has been placed on routine surveillance protocol which includes a follow-up visit to the Oncology outpatient department once every 6 months for the first 2 years and then once every one year for next 3 years. At the time of the visit the patient will undergo a whole body FDG PET-CT scan, a 2D Echocardiogram (to look for cardiotoxic side effects of Adriamycin), and routine laboratory investigations. Till date the patient is in clinical remission as documented on the last outpatient department visit. The patient is alive and asymptomatic without disease progression for the last twenty months after initial diagnosis, without any evidence of local or systemic recurrence.

## 3. Discussion

Lymphoma, malignant monoclonal proliferation of lymphoid cells, is the second most frequent malignant tumor (incidence 2.5%) in the head and neck region [8, 9]. Most primary lymphomas of the head and neck are of the NHL variety and are generally extranodal in location at initial presentation occurring most commonly in the nasopharynx, lacrimal sac, and the temporal bone [10]. Primary lymphoma of the temporal bone involves the mastoid, middle ear most commonly, and the external/internal auditory canals to a lesser degree [6]. A layer of lymphoid tissue located deep to the epithelium of the mucosa lining the mastoid antrum, tympanic cavity, and tympanic orifice of the eustachian tube acts as the site of origin of the primary lymphoma. Bilateral affection by primary B-cell NHL involving mastoids without middle ear cavity affection is extremely uncommon and to the best of our knowledge the imaging features of this entity have not been reported in medical literature. Osteolytic permeative lesions involving temporal bones should be viewed with a high degree of suspicion for malignancy as similar appearances in adults can be seen with multiple myeloma, metastatic pathology, and aggressive infective pathologies like fulminant coalescent mastoiditis (especially in diabetic patients) and mycosis fungoides [11]. Differentiation based on clinical presentation may be difficult at times as patients present with similar complaints like ear pain, swelling in pre- and postauricular regions, and facial palsy [12–14]. Facial nerve palsy is rare in lymphoma because the nerve sheath is resistant to tumor invasion. Nerve palsy occurs when the bony facial canal is destroyed by the tumor and nerve fibers are infiltrated by the tumor cells [15]. Affection of the facial nerve by the tumor is usually seen in the region of the geniculate ganglion [14]. In this particular case, the left facial nerve infranuclear palsy was due to erosion of the bony descending mastoid segment of the left intratemporal facial nerve canal and direct nerve involvement by the tumor mass at and distal to the stylomastoid foramen. HRCT and contrast enhanced MRI scans are essential for a comprehensive evaluation of malignancies affecting the temporal bone. HRCT depicts bony details including type of bone destruction, status of middle ear cavity involvement, ossicular chain evaluation, integrity of the tegmen tympani, and exact site/extent of the intratemporal bony facial canal involvement. MRI is useful in identifying leptomeningeal and brain parenchymal invasion as well as mapping the entire extent of extracranial soft tissue involvement. Contrast enhanced MRI scans also provide information about perineural spread on the facial nerve [16]. PET-CT is essential to stage the disease, document pathology elsewhere in the body, and assess response to therapy. Biopsy and immunohistochemistry help in planning a cell targeted chemotherapeutic regime.

## 4. Conclusion

Our case report highlights the need to have a high degree of suspicion for malignancy even in bilateral temporal bone pathologies (which are more commonly seen in infective conditions). Primary lymphoma of the temporal bone, though rare, should be considered in the differential diagnosis of patients presenting with ear related symptoms and associated facial nerve palsy especially in the elder age group. HRCT and contrast enhanced MRI are essential to comprehensively evaluate such cases in order to effectively plan further management. As there may not be specific imaging criteria to make a definitive diagnosis, this can only be confirmed by histopathology and immunohistochemistry. PET-CT is useful in staging the disease and scrupulous surveillance is needed to monitor response to therapy and to identify early signs of tumor recurrence.
